# Is lymphadenectomy a prognostic marker in endometrioid adenocarcinoma of the human endometrium?

**DOI:** 10.1186/1471-2407-10-224

**Published:** 2010-05-21

**Authors:** Nina Bassarak, Thomas Blankenstein, Ansgar Brüning, Darius Dian, Florian Bergauer, Klaus Friese, Ioannis Mylonas

**Affiliations:** 1 1st Department of Obstetrics and Gynaecology, Campus Innenstadt, Ludwig-Maximilians-University Munich, Munich, Germany

## Abstract

**Background:**

During surgery for endometrial cancer, a pelvic lymphadenectomy with or without para-aortic lymphadenectomy is performed at least in patients with risk factors (stage I, grading 2 and/or histological subtypes with higher risk of lymphatic spread), and is hence recommended by the International Federation of Obstetrics and Gynecology (FIGO). Although lymph node metastases are important prognostic parameters, it has been contentious whether a pelvic lymph node dissection itself has a prognostic impact in the treatment of endometrial cancer, especially in endometrioid adenocarcinoma. Therefore, this study evaluated whether lymphadenectomy has a prognostic impact in patients with endometrioid adenocarcinoma.

**Methods:**

The benefits of lymphadenectomy were examined in 214 patients with a histological diagnosis of endometrial adenocarcinoma. Tumour characteristics were analysed with respect to the surgical and pathological stage.

**Results:**

Of the 214 patients with endometrial adenocarcinoma, 171 (79.9%) were classified as FIGO stage I, 15 (7.0%) FIGO stage II, 21 (9.8%) FIGO stage III and 7 (3.3%) FIGO stage IV. One hundred and thirty four (62.6%) of the patients had a histological grade 1 tumour, while 56 (26.2%) and 24 (11.2%) had a histological grade 2 or grade 3 tumour, respectively. Lymphadenectomy was performed in 151 (70.6%) patients. Only 11 (5.1%) patients showed metastatic disease in the lymph nodes. The performance of a lymphadenectomy resulted in significantly increased cause-specific and overall survival, while progression-free survival was not affected by this operative procedure.

**Conclusions:**

The performance of an operative lymphadenectomy resulted in better survival of patients with endometrioid adenocarcinoma. This increase was significant for cause-specific and overall survival, while there was a tendency only towards increased progression-free survival. Therefore, even in endometrioid adenocarcinoma, a pelvic and/or para-aortic lymphadenectomy should be performed.

## Background

Endometrial cancer is the most common female pelvic malignancy in industrial countries, with an estimated annual incidence of 15-20 per 100,000 women. The lifetime risk of developing endometrial cancer is approximately 2.5%, while the lifetime probability of dying from this cancer is estimated at 0.52% [1,2]. In 1988, the International Federation of Gynecology and Obstetrics (FIGO) established a new pathological and surgical staging system for endometrial cancer. The surgical staging includes an explorative laparatomy with radical hysterectomy and bilateral salpingo-oophorectomy, peritoneal washing, and pelvic and para-aortic lymph node sampling [3]. However, no specifications regarding the type and extent of pelvic lymph node dissection have been established. The extent (sampling or dissection) and performance of additional para-aortic sampling varies from surgeon to surgeon [4]. Particularly in patients with a low risk for lymph node metastases (due to superficial depth of invasion, small size of tumour and low tumour grade) a routine lymph node dissection is often not performed. Moreover, substantial co-morbidities such as obesity or old age are considered contraindications to a full pelvic and/or para-aortic lymphadenectomy. Therefore, pelvic and/or para-aortic lymph node dissection has been a subject of continuous debate.

In 1987 and 1991, two large staging trials identified important prognostic factors in endometrial carcinoma, primarily age, race and endocrine status [5,6]. Uterine factors included histological cell type, tumour grade, depth of myometrial invasion, occult extension of the cervix and vascular space invasion. Additionally, extrauterine prognostic factors were defined as adnexal metastasis, other extrauterinal spread, positive peritoneal cytology, pelvic lymph node metastases, and para-aortic involvement [5,6]. However, it was not assessed whether a lymphadenectomy itself has prognostic value in patients with endometrial cancer. Since then, a few studies have addressed this issue, but the results and conclusions have been rather inconsistent. In particular, the value of lymphadenectomy in early stage cases is still controversial [7-9]. Some recent studies have investigated the impact of the extent of lymph node removal in surgical treatment of endometrial cancer. The results demonstrated an impact on survival for patients with a higher number of dislodged lymph nodes, especially in cases showing high-risk clear-cell or serous-papillary histology [10-12]. In 2007, a retrospective analysis by the Surveillance Epidemiology and End Results program (SEER) in the U.S.A. reviewed a large population database and concluded that lymphadenectomy results in improved survival of patients with endometrial cancer [13]. However, in a recent prospective study, there was no evidence of benefit from pelvic lymphadenectomy in terms of overall or recurrence-free survival in women with early endometrial cancer [14].

The aim of this study was to evaluate the outcome of patients with endometrioid adenocarcinoma, histologically the most common type of endometrial cancer (approximately 80% of all endometrial cancers) [15], with regard to performance of a lymphadenectomy in a well-characterized cohort population. The study also aimed to assess whether performance of a lymph node dissection constitutes an independent prognostic factor in this patient group. Finally, we examined whether performance of a lymphadenectomy constitutes a prognostic parameter in early-stage compared to late-stage disease.

## Methods

For this retrospective study, we reviewed pathological and surgical records of 308 patients operated on between 1990 and 2002 in the 1^st ^Department of Obstetrics and Gynaecology, Campus Innenstadt, Ludwig-Maximilians-University Munich. This patient group had been previously well characterised and several prognostic markers had been evaluated [16,17]. All patients had a preoperative ultrasound imaging examination and underwent hysterectomy, bilateral salpingo-ophorectomy, and optional pelvic and/or para-aortic lymph node sampling. The decision to perform lymphadenectomy was made by the surgeon and was influenced by the patients' characteristics (histological report of abrasion, progress of cancer, age, obesity and further comorbidities) as well as rapid intraoperative histological evaluation. Pathological reports were examined for histologic type, FIGO stage, grade, depth of myometrial invasion and lymph node status. Pathological stage, histological subtype and lymph node status were defined for each surgical specimen according to the 1988 FIGO criteria [3]. Histological classification was made according to the World Health Organization (WHO) system as well-differentiated (G1), moderately-differentiated (G2) and poorly-differentiated (G3). Only patients with endometrioid adenocarcinoma histology were included. Exclusion criteria were serous-papillary, clear-cell, pure squamous or mesenchymal histology, or mixed histology and pre-operative radiation.

Patient data were supplemented with medical records from the oncology archives of our hospital, and the Munich tumour registry. The Munich tumour registry systematically collects baseline data, including demographic data, diagnosis, additional diseases (e.g., obesity, diabetes or hypertension) and treatment information for all cancer patients who are diagnosed or treated in the 1^st ^Department of Obstetrics and Gynaecology. Automated records and, when available, charts for each patient were reviewed to verify diagnosis and the presence or absence of radiologic or pathological evidence of disease recurrence. Patient data were analyzed anonymously. For all cases of recurrence there was radiologic evidence or biopsy-proven progression of disease. Only the records of patients who died of disease were considered to be uncensored; the records of all patients who were alive at follow-up or who did not die of disease (or a related cause) were considered to be censored. Additionally, those cases where the exact cause of death was unknown but the patient died within two years after diagnosis of a metastatic lesion have been censored as previously described [16,17]. The surveillance period of patients was variable and ranged from 3 days to 176 months, with a median of 86 months.

### Statistical analysis

Pearson χ^2 ^and Fisher's exact test were used for categorical variables where applicable. The outcome variables analyzed were progression-free survival, cause-specific survival and overall survival. Univariate analysis was performed with Kaplan-Meier life-table curves to estimate survival and were compared using the log-rank test [18]. Prognostic models used the Cox regression analysis for multivariate analyses of survival in a forward stepwise manner, as previously described [16,17,19]. Variables tested in this multivariate analysis were age at surgery, FIGO stage, grading, lymphangiosis, myometrial invasion, cervical involvement, ovarian metastasis, adjuvant radiotherapy, occurrence of lymph node metastasis and the performance of lymphadenectomy. Hazard ratios (HR), 95% confidence intervals (CI) and *p*-values are reported. Significance of differences was assumed at *p *≤ 0.05 at the two-sided tests (SPSS, version 15.0; SPSS Inc., Munich, Germany).

## Results

### Clinical and pathological characteristics

The clinical and pathological characteristics of the patients with endometrioid adenocarcinoma are summarized in Table 1. Only patients (n = 214) with endometrioid histology were included in this analysis. Mixed and mucinous adenocarcinomas were also excluded from analysis. The percentile distribution of stage I to IV disease was comparable to the data provided in the international literature [20], demonstrating the validity of the patient group enrolled in this analysis. A lymphadenectomy was performed in 151 (70.6%) of these patients. Pathological investigation revealed that the lymph nodes were positive for disease in 11 (5.1%) of analysed cases. The median patient age at time of surgery was 65.1 years (range 35.5-87.9). Lymphadenectomy was performed in 90 (84.1%) patients younger than 65 years and 61 (57.0%) patients older than 65 years.

**Table 1:** Clinical and pathological characteristics.

		Total	No Lymphadenectomy	Lymphadenectomy	p-Wert (Chi2)
**Patient number**		n = 214	n = 63 (29.4%)	n = 151 (70.6%)	

**Age at surgery**	*>65 years*	107 (50%)	46 (43.0%)	61 (57.0%)	<0.001
	*≤65 years*	107 (50%)	17 (15.9%)	90 (84.1%)	

**FIGO Stage**	*I*	171 (79.9%)	51 (29.8%)	120 (70.2%)	N.S.
	*II*	15 (7.0%)	6 (40.0%)	9 (60.0%)	
	*III*	21 (9.8%)	3 (14.3%)	18 (85.7%)	
	*IV*	7 (3.3%)	3 (42.9%)	4 (57.1%)	

**WHO Grade**	*1*	134 (62.6%)	36 (26.9%)	98 (73.1%)	N.S.
	*2*	56 (26.2%)	18 (32.1%)	28 (67.9%)	
	*3*	24 (11.2%)	9 (37.5%)	15 (62.5%)	

**Lymphangiosis**	*Negative*	197 (92.1%)	57 (28.9%)	140 (71.1%)	N.S.
	*Positive*	17 (7.9%)	6 (35.3%)	11 (64.7%)	

**Deep myometrial invasion**	*only endometrium*	34 (15.9%)	9 (26.5%)	25 (73.5%)	N.S.
	*<50%*	111 (51.9%)	31 (27.9%)	80 (72.1%)	
	*>50%*	69 (32.2%)	23 (33.3%)	46 (66.7%)	

**Cervical involvement**	*negative*	191 (89.3%)	55 (28.8%)	136 (71.2%)	N.S.
	*positive*	23 (10.7%)	8 (34.8%)	15 (65.2%)	

**Ovarian metastasis**	*negative*	204 (95.2%)	61 (29.9%)	143 (70.1%)	N.S.
	*positive*	10 (4.7%)	2 (20%)	8 (80%)	

**Obesity**	*Negative*	137 (64.0%)	39 (28.5%)	98 (71.5%)	N.S.
	*Positive*	77 (36.0%)	24 (31.2%)	53 (68.8%)	

**Diabetes**	*Negative*	187 (87.4%)	49 (26.2%)	138 (73.8%)	<0.05.
	*Positive*	27 (12.6%)	14 (51.9%)	13 (48.1%)	

**Hypertension**	*Negative*	126 (58.49)	25 (19.8%)	101 (80.2%)	<0.001.
	*Positive*	88 (41.1%)	38 (43.2%)	50 (56.8%)	

**Adjuvant radiotherapy**	*Not performed*	138 (64.5%)	43 (31.2%)	95 (68.8%)	N.S.
	*performed*	76 (35.5%)	20 (26.2%)	56 (73.3%)	

Most of the patients (n = 171 (79.9%) were diagnosed at FIGO stage I (stage IA: 29 (13.6%), stage IB: 99 (46.3%), stage IC: 42 (20.1%)), and 120 (70.2%) patients underwent lymphadenectomy. Of these patients, 81 (67.5%) presented with histological differentiation grade I, 29 patients (24.2%) with grade II and 10 (8.4%) with grade III. There was no difference in grading when compared to the non-lymphadenectomy group. Thirty-three patients (64.7%) showed a WHO histological grade 1, while 15 patients (29.4%) and 3 patients (5.9%) showed grade 2 and grade 3, respectively (*p *> 0.05).

Fifteen (7.0%) patients were classified as FIGO stage II (stage IIA: 2 (0.9%), stage IIB: 13 (6.12%)), and 60% of these patients underwent lymphadenectomy. There were 21 (9.8%) patients with FIGO stage III cancer (stage IIIA: 9 (4.2%), stage IIIB: 3 (1.4%) stage IIIC: 9 (4.2%)), and the lymph nodes were removed in 85.7% of these cases. The lymph nodes were positive for disease in all the FIGO stage III patients, and these patients were scaled to this stage after the operation, as suggested by the FIGO classification system [3]. A lymphadenectomy was performed in four out of the seven (3.3%) FIGO stage IV patients.

One hundred and thirty four (62.6%) patients presented with a well-differentiated histology (WHO grade 1), and a lymphadenectomy was performed in 98 (73.1%) of these patients. The lymph nodes were removed in 67.9% of the 56 (26.2%) patients with a grade of moderate differentiation. The number of patients with poorly-differentiated histology was 24 (11.2%), and 62.5% of these patients underwent lymphadenectomy.

Seventeen of the 214 analyzed patients had a lymphangiosis, and six patients exhibited a haemangiosis. The frequency of lymphadenectomy was slightly lower in patients with lymphangiosis (64.7%) or haemangiosis (66.7%) compared to patients without (71.1% and 70.7%).

Obesity was observed in 77 (36.0%) cases. Although lymphadenectomy is more difficult in obese patients, the frequency of lymphadenectomy in these patients (68.8%) was in fact similar to that in non-obese patients (71.5%). Of the 214 patients, 76 (35.5%) received adjuvant radiotherapy. The lymph nodes were removed in 73.3% of these patients, compared to 68.8% in patients without adjuvant radiotherapy. Anti-hormonal therapy was administered to seven (3.3%) patients, and a lymphadenectomy was performed in three of these patients. Among the factors examined for association with lymphadenectomy, significant differences were found only for age, diabetes and hypertension (Table 1).

### Survival analyses

Recurrence or progression was observed in 29 (13.6%) patients, while 62 (29.0%) patients died during follow-up, and 25 (11.68%) patients died as a result of the endometrial cancer. The median follow-up time for all uncensored patients was 98.7 months (range 0.3-176.8 months), and the median time to death in the uncensored subgroup was 55.3 months (range 0.72-143.0 months).

**Figure 1:**
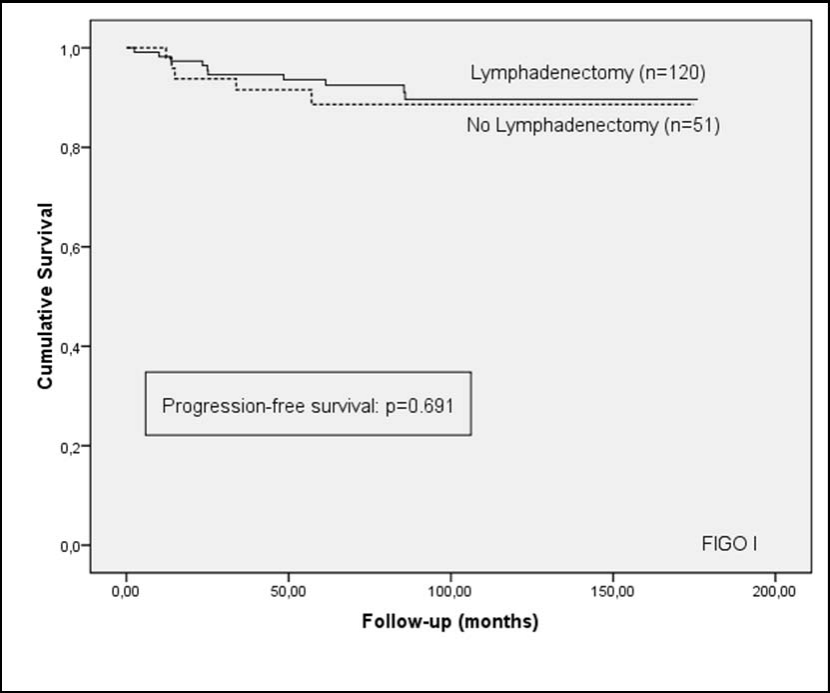
**Progression-free survival**. Kaplan-Meier curve of clinical outcome for progression-free survival demonstrates no significant difference arising from lymphadenectomy for patients at FIGO stage I **(A)**, or all stages **(B)**. Follow-up time is defined as the time until progression occurred.

**Figure 2:**
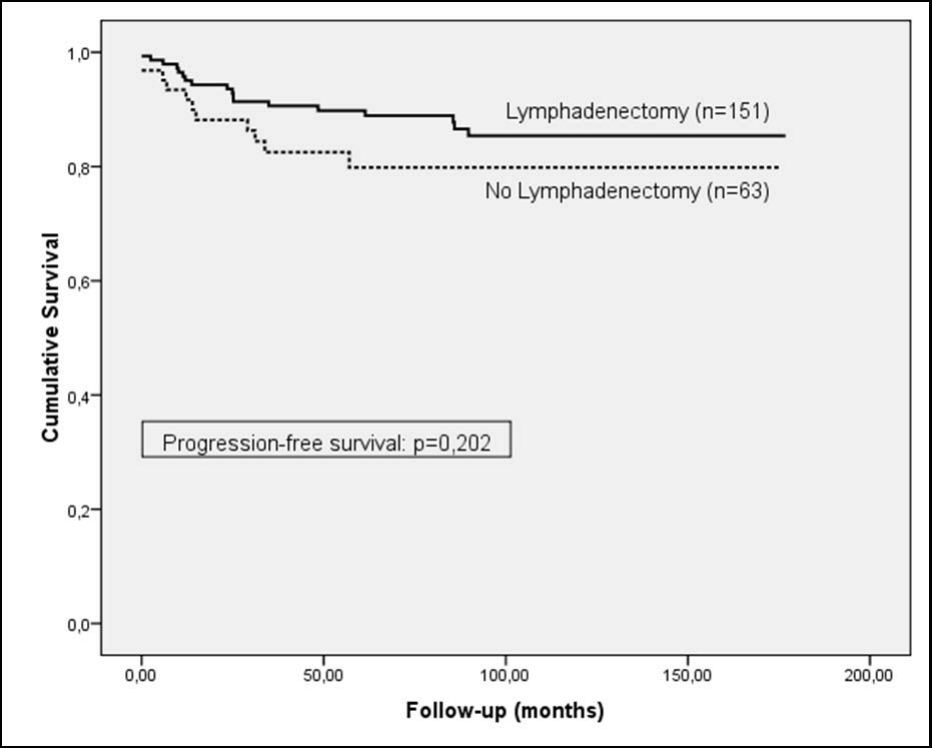
**Cause-specific survival**. Kaplan-Meier curve of clinical outcome for cause-specific survival demonstrates a significant impact on patients treated with lymphadenectomy (log-rank: *p = *0.044) at early FIGO stage **(A) **and all stages **(B)**. Follow-up time is defined as the time until cause-specific death occurred.

**Figure 3:**
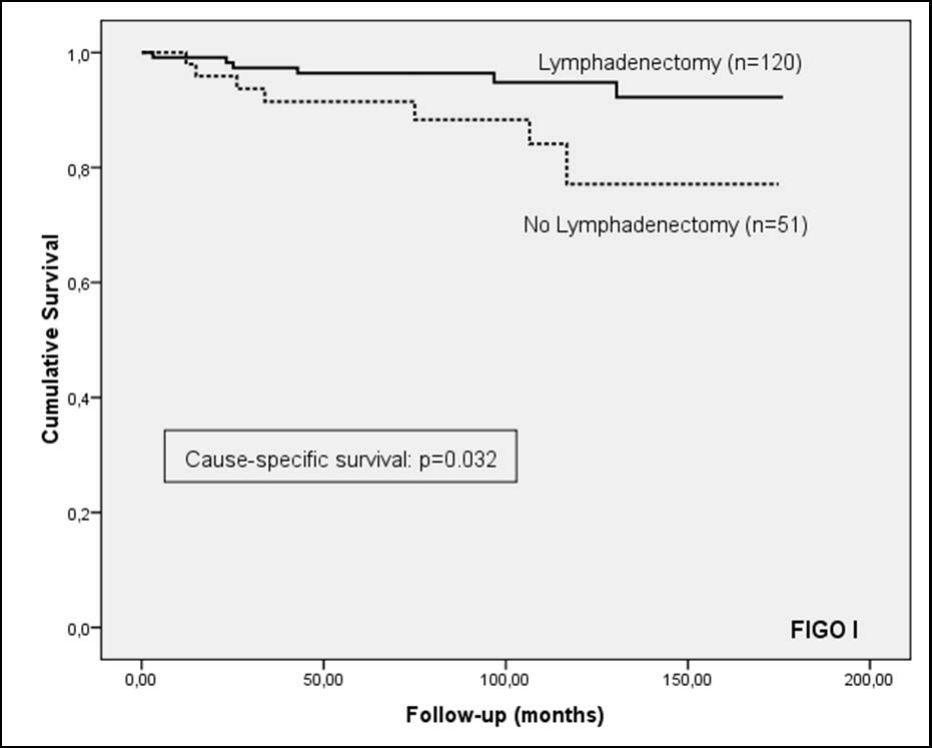
**Overall survival**. Kaplan-Meier curve of clinical outcome for overall survival demonstrates a significant impact on patients treated with lymphadenectomy for FIGO stage I patients **(A) **and all stages **(B) **(log-rank: *p *< 0.001). Follow-up time is defined as the time until death occurred.

**Figure 4:**
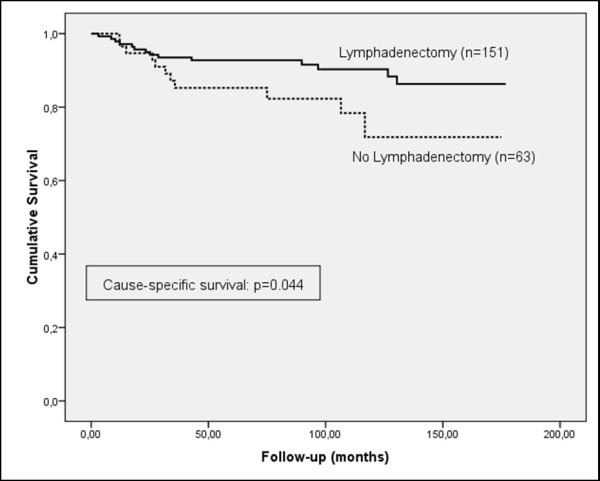
**Cause-specific survival for patients at all stages**. Kaplan-Meier curve of clinical outcome for cause-specific survival demonstrates a significant impact on patients treated with lymphadenectomy (log-rank: p=0.044) at all stages. Follow-up time is defined as the time until cause-specific death occurred.

**Figure 5:**
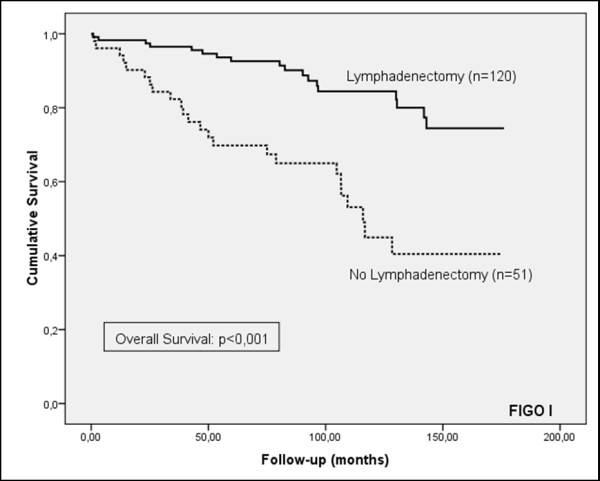
**Overall survival for patients at FIGO stage I**. Kaplan-Meier curve of clinical outcome for overall survival demonstrates a significant impact on patients treated with lymphadenectomy for FIGO stage I patients (log-rank: p<0.001). Follow-up time is defined as the time until death occurred.

**Figure 6:**
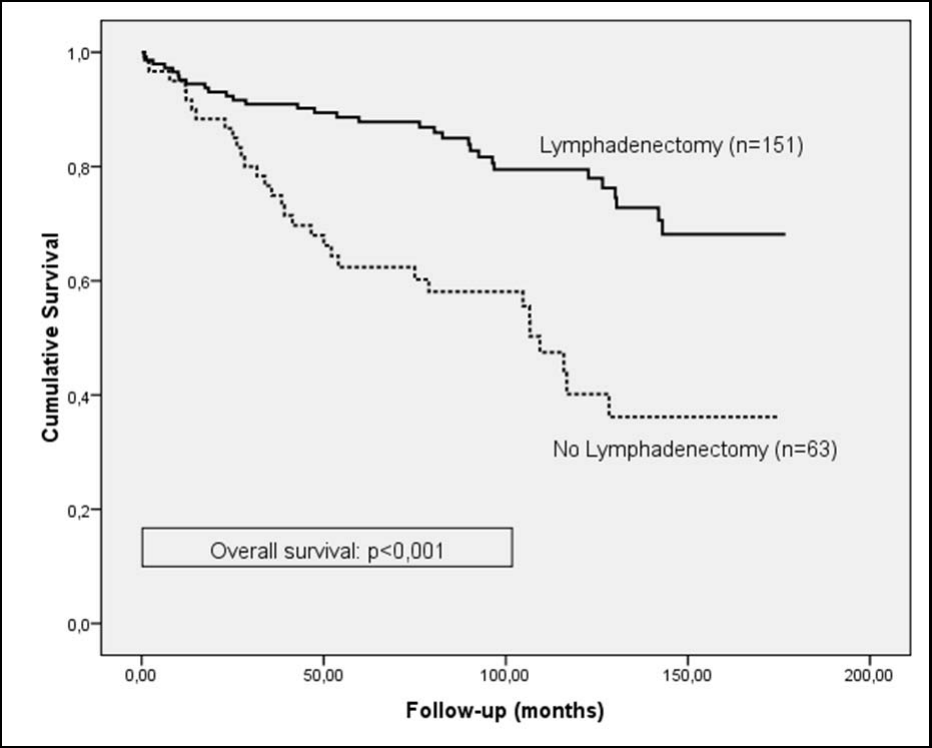
**Overall survival for patients at all stages**. Kaplan-Meier curve of clinical outcome for overall survival demonstrates a significant impact on patients treated with lymphadenectomy for all stages (log-rank: p<0.001). Follow-up time is defined as the time until death occurred.

Although the frequency of lymphadenectomy was higher in patients who did not suffer a recurrence of the cancer (71.9%, versus 62.1% in patients who did suffer a recurrence) and the progression-free survival curve showed a tendency to increased survival for patients who underwent lymphadenectomy, the results were not statistically significant for patients in the early stage (FIGO I), or for all stages (Figures 1 and 2). However, a significant prognostic benefit in terms of increased cause-specific survival was observed for patients who had their lymph nodes removed, for patients in early-stage (FIGO I), and for all stages (Figures 3 and 4). The overall survival curve for all analysed cases also showed a significant prognostic impact of lymphadenectomy (*p *=< 0.001) (Figures 5 and 6).

### Multivariate Cox regression analysis

Multivariate Cox regression analysis was performed to evaluate lymphadenectomy as an independent prognostic marker in patients with endometrioid adenocarcinoma (Table 2). The following well known parameters influencing survival were included: age, FIGO stage, WHO grading, cervical involvement, deep myometrial invasion, ovarian metastasis, presence of positive nodes, and adjuvant radiotherapy. For overall survival, a significant effect was observed for age (*p *< 0.001), FIGO stage (*p *= 0.002), grading (*p *= 0.016), cervical involvement (*p *< 0.001) and lymphadenectomy (*p *= 0.001). Cause-specific survival was influenced by FIGO stage (*p *= 0.006), cervical involvement (*p *= 0.010), deep myometrial invasion (*p *= 0.019) and lymphadenectomy (p = 0.005). For progression-free survival, FIGO stage (*p *< 0.001), grading (*p *= 0.041), cervical involvement (*p *= 0.019) and lymphadenectomy (*p *= 0.022) were significant prognostic factors.

**Table 2:** Hazard ratios by multivariate Cox regression analysis.

	Progression-free survival			Cause-specific survival			Overall survival		
	HR	CI (5-95%)	p	HR	CI (5-95%)	p	HR	CI (5-95%)	p
**Age (>65 years)**	-	-	-	-	-	-	3.779	2.006 - 7.118	***<0.001***
**WHO Grading (G 1/2 vs. G 3)**	2.631	1.038 - 6.667	***0.041***	-	-	-	2.214	1.158 - 4.231	***0.016***
**FIGO Stage (1/2 vs. 3/4)**	7.827	3.176 - 19.288	***<0.001***	3.870	1.461 - 10.254	***0.006***	2.874	1.460 - 5.655	***0.002***
**Cervical involvement**	2.861	1.190 - 6.878	***0.019***	3.403	1.333 - 8.690	***0.010***	3.456	1.742 - 6.856	***<0.001***
**Deep myometrial invasion**				3.172	1.461 - 10.254	***0.019***			
**Performed Lymphadenectomy**	0.376	0.162 - 0.871	***0.022***	0.308	0.134 - 0.706	***0.005***	0.403	0.234 - 0.695	***0.001***

## Conclusions

Endometrial cancer is the most common female pelvic malignancy in the western world. Endometrioid histology is thought to be a low-risk histology with a very good prognosis, especially if diagnosed early [15]. The five-year survival rate is dependent on stage, and is 87-93% for grade 1 and 2 and approximately 61% for poorly-differentiated endometrioid adenocarcinomas. In contrast, the five-year survival rate for clear-cell adenocarcinoma is approximately 42%, and only 24-34% for cases of serous-papillary histology [21].

Staging and therapeutic surgical intervention includes an explorative laparatomy with radical hysterectomy and bilateral salpingo-oophorectomy, peritoneal washing, and pelvic and para-aortic lymph node sampling [3]. However, no specifications regarding the type, extent and prognostic impact of pelvic lymph node dissection have been established, this being a controversial topic of ongoing debate.

In 1995, Kilgore and colleagues published a retrospective analysis of 649 patients with adenocarcinoma of the endometrium. Of these patients, 212 underwent multiple-side lymph node sampling and 205 patients had limited pelvic node sampling (less than four pelvic sites), while 208 patients were not sampled. Overall survival was significantly better in patients with multiple-site lymph node sampling. Adjuvant radiation or splitting into low- and high-risk groups did not affect this result [7].

In a 2007 analysis of the large SEER database from the United States, 42,814 patients with endometrial adenocarcinoma were analysed by multivariate Cox regression analysis [13], and the rate of lymphadenectomy was found to be only 46%. Lymphadenectomy was identified as an independent prognostic factor for overall survival, with hazard ratios of 0.74 for the subgroup with more than 11 lymph nodes dissected, and 0.89 for the subgroup with 1-11 nodes removed [13].

Although a very small number of studies have not shown that lymphadenectomy has an impact on survival [9], most studies have demonstrated increased survival and have established lymphadenectomy as an important prognostic factor. This is also supported by the results of a few authors who have detected an increasing frequency of lymphadenectomy in recent decades. For example, in a retrospective analysis of 1,312 patients, Barakat *et al. *demonstrated an increase from 28% in 1993 to 82% in 2004 [22]. For patients of the SEER program, the frequency has risen from 31% in 1988 to 53% in 2003 [13].

However, the relevance of overall survival is limited, because compared to other patients, the frequency of lymphadenectomy is low in older patients with comorbidities. For this reason, analysis of progression-free survival is a more meaningful approach. In our study, the results from the progression-free survival curve are not statistically significant. However, the results from multivariate analysis (*p *= 0.016) argue that lymphadenectomy does have a major influence on progression-free survival. Since patients with FIGO stage I also did not show increased survival, we assume that the usefulness of lymphadenectomy as an independent prognostic factor results from increased survival of patients in higher stages. In this study, the number of patients with grade 3 (11.2%) or higher (13.1%) FIGO stage cancer is very low, so it is not possible to perform adequate statistical analysis of survival. Nevertheless, we were able to demonstrate that lymphadenectomy had an impact on cause-specific and overall survival of patients with endometrioid histology, for patients of early-stage, and all stages.

The question of whether pelvic lymphadenectomy improves survival rates of high-risk patients has not yet been resolved. Lutman *et al. *demonstrated improved overall and progression-free survival in a group of patients with FIGO stage 1-2, high-risk histology (clear-cell, papillary serous or grade 3 endometrioid histology) and more than 12 lymph nodes dissected. For low-risk cases, the results were not significant [11]. Similarly, in another study no significant impact on overall survival was demonstrated for low risk-patients (grade 1 and 2, outer-half myometrial invasion, no serous-papillary or clear-cell differentiation) [12]. Further, in a recent prospective study, there was no evidence of benefit in terms of overall or recurrence-free survival arising from pelvic lymphadenectomy in women with early endometrial cancer [14].

A contribution to the survival of patients who undergo lymphadenectomy may be the identification of patients with nodes positive for disease, a significant prognostic parameter [5], and the administration of a suitable adjuvant therapy. However, the incidence of cases with nodal metastases is very low, which suggests that lymphadenectomy itself has a therapeutic benefit. A final possible explanation is the removal of occult small metastatic disease which was not detected by classical histopathological evaluation [23,24].

There is a small number of unsolved issues. The extent of the lymphadenectomy would have a major impact on the benefit to patient survival, meaning that as more lymph nodes were removed, the probability would increase of identifying patients with nodes positive for disease. Also, the therapeutic effect of removing occult lymph node metastases would rise. For this analysis, we disregarded the extent and localisation of the removed lymph nodes. In the literature, the cut-off for splitting patients undergoing lymphadenectomy into a limited sampling and a multiple sampling group is around 10 to 12 lymph nodes. Although this number is essentially the median, it is basically a randomly-selected range, and exceeding the cut-off does not mean that a full dissection will be made. Of course, the regions sampled also play a decisive role in the patients' outcome. An impact on survival of para-aortic sampling in patients with high-risk adenocarcinoma of the endometrium was demonstrated by Chang and colleagues [25]. However, it is not yet known whether para-aortic lymph node dissection could play a substantial role in routine treatment of all patients. Furthermore the possibility of underestimating FIGO IIIC disease in non-surgically-staged patients remains, and this problem cannot be solved by any current imaging modality.

The main purpose of this analysis was to investigate the effect of lymphadenectomy only in patients with endometrioid histology. Our results show that lymphadenectomy improves patient survival, and so demonstrate the prognostic relevance of this intervention in treatment of patients with endometrioid adenocarcinoma. Therefore, a pelvic and/or para-aortal lymphadenectomy should be performed during surgery, even in endometrioid adenocarcinoma. In a recent prospective study, there was no evidence of benefit in terms of overall or recurrence-free survival from pelvic lymphadenectomy in women with early endometrial cancer [14]. However, these results remain to be confirmed in further studies.

## Competing interests

The authors declare that they have no competing interests.

## Authors' contributions

NB and TB carried out the data collection and data analysis, and drafted the manuscript. AB participated in the design of the study, performed the statistical analysis and participated in the interpretation of data. DD and FB participated in the design of the study and the acquisition and interpretation of data. FK was involved in the interpretation of data and critically revised the manuscript. IM conceived the study, participated in its design and coordination, and helped in statistical analysis and drafting of the manuscript. All authors have read and approved the final manuscript.

## Pre-publication history

The pre-publication history for this paper can be accessed here:

http://www.biomedcentral.com/1471-2407/10/224/prepub
